# A Plan-Do-Study-Act Approach to the Development, Implementation and Evaluation of a Patient Navigation Program to Reduce Breast Cancer Screening Disparities in Un- and Under-Insured, Racially and Ethnically Diverse Urban Women

**DOI:** 10.3389/fpubh.2021.595786

**Published:** 2021-02-19

**Authors:** Carmen E. Guerra, Emily Verderame, Andrea Nicholson, LiYea Wan, Ari D. Brooks

**Affiliations:** ^1^Department of Medicine, Perelman School of Medicine, University of Pennsylvania, Philadelphia, PA, United States; ^2^Abramson Cancer Center, University of Pennsylvania, Philadelphia, PA, United States; ^3^Leonard Davis Institute for Health Economics, University of Pennsylvania, Philadelphia, PA, United States; ^4^MD Anderson Cancer Center at Cooper, Cooper University Hospital, Camden, NJ, United States; ^5^Department of Surgery, Perelman School of Medicine, University of Pennsylvania, Philadelphia, PA, United States

**Keywords:** breast cancer screening, neoplasm, patient navigation, Plan-Do-Study-Act, uninsured, low SES

## Abstract

**Introduction:** For the over 28 million Americans without health insurance, there is a great need to develop programs that help meet the health needs of the uninsured population.

**Materials and Methods:** We applied the Plan-Do-Study-Act (PDSA) quality improvement framework to the development, implementation, and evaluation of a breast cancer screening navigation program for un- and under-insured women.

**Results:** Six critical steps emerged: (1) obtain program funding; (2) navigator training; (3) establish a referral base network of community partners that serve the un- and under-insured women; (4) implement a process to address the barriers to accessing mammography; (5) develop a language- and culturally-tailored messaging and media campaign; and (6) develop measures and process evaluation to optimize and expand the program's reach.

**Discussion:** A Plan-Do-Study-Act approach allowed identification of the key elements for successful development, implementation and optimization of a breast cancer screening navigation program aimed at reaching and screening un- and underinsured women.

## Introduction

Breast cancer is the most common cancer and second leading cause of cancer death among women in the United States (U.S.). Despite a reduction in breast cancer mortality by 40% from 1989 to 2016, breast cancer continues to pose a significant public health burden (1). It is estimated that in 2020, there will be approximately 276,480 cases diagnosed in women and about 42,170 deaths due to breast cancer in the U.S. (1). Furthermore, the rate of decline in death rates has not been equitable. Breast cancer death rates are approximately 40% higher in Black women compared to white women, despite similar incidence rates (2). Racial and ethnic minority groups in the U.S are more likely to be medically underserved and live in poverty compared to their White counterparts (2). Racial disparities in breast outcomes exist due to social, economic, and cultural factors such as socioeconomic status, employment status; and limited access to healthcare, safe housing and affordable nutritious food (2). Although breast cancer screening reduces breast cancer mortality and thereby remains the cornerstone of breast cancer control (3), screening remains underutilized in the U.S. (4). In 2018, 73% of women aged 50–74 years reported having had a mammogram within the past 2 years (5). However, according to the Center for Disease Control (CDC), several subpopulations of patients have lower screening rates (6). In particular, only 30% of uninsured women over age 40 had undergone a screening mammography within the past 2 years compared to 69.7% of insured women (6). Uninsured women are also 2.6 times more likely to be diagnosed at a later stage of disease and 60% more likely to die from breast cancer compared to women with health insurance (7). While there have been recent improvements in access to insurance in the U.S., 28.5 million individuals living in the U.S., or 8.5% of the population, did not have health insurance at any point during 2018 (8). Regardless of race/ethncity, for Americans without health insurance, there is a great need to develop programs that help meet the health needs of the uninsured population.

Patient navigation programs have been reported to reduce barriers to care and improve access to services. Navigation programs have also been shown to improve the quality measures including increasing the receipt of timely screening and diagnostic services and treatment after a suspicious finding, adherence to treatment, and patient satisfaction (9). Furthermore, such programs are cost-effective (9). In order to encourage others to design and implement navigation programs in diverse, un- and under-insured communities that meet the needs of their communities, we describe the use of the Plan-Do-Study-Act (PDSA) Framework, first proposed by W. Edwards Deming and popularized by the Institute for Health Care Improvement (10), to the process of developing a breast cancer screening navigation program for un and under-insured women, its key successes, as well as the challenges and how these challenges were overcome. This project and all procedures performed in studies involving human participants were in accordance with the ethical standards and approved by the University of Pennsylvania's Institutional Review Board (IRB).

## Materials and Methods

Below we describe the development of the Penn Medicine Breast Health Initiative (PBHI), a breast cancer screening navigation program, created to increase access to free, high-quality mammography for un- and underinsured women and reduce breast cancer mortality among this underserved group, using the PDSA framework. The Initiative is based at the Abramson Cancer Center (ACC) of the University of Pennsylvania, a National Cancer Institute Comprehensive Cancer Center, located in Philadelphia. The ACC is also home to the Rena Rowan Breast Center and the Pennsylvania Hospital Integrated Breast Center which are accredited by the American College of Surgeons National Accreditation Program for Breast Centers. We first describe the needs assessment that led to the program's creation, then apply the PDSA model to establishing, evaluating and optimizing the program.

### Needs Health Assessment

Understanding the health need from the perspective of the community is the critical step to designing a successful navigation program. It informs the purpose and goals of the navigation program. Similar to national data (6), data from the Public Health Management Corporation's Southeastern Pennsylvania Household Survey indicated that, in 2015, nearly four in ten (38%) uninsured women in Philadelphia between the ages of 50–74 reported not having a mammogram in the past year; this figure represents an estimated 462,200 women (11). The lower screening rates among uninsured women contribute to Philadelphia's breast cancer mortality rate of 28.2/100,000 which is significantly higher than the state and national rates of 22.8/100,000 and 21.5/100,000, respectively (12). The un- and under-insured population in Philadelphia, thus, represented one of the populations with the greatest need for interventions to increase access to screening mammography.

### Plan-Do-Study-Act Framework

There are multiple steps to developing a successful patient navigation program (13). Using the Plan-Do-Study-Act (PDSA) Framework (10), we discuss several critical components to the development of a breast cancer screening patient navigation program for diverse, un- and under-insured populations. In the “Plan” stage, the critical elements are: (1) securing the funding to establish a cost-free screening mammography program; (2) selection, training and defining the functions of the breast cancer screening navigator; and (3) establishment of a network of community partners that serve the un and under-insured and provide a referral base. The “Do” stage is focused on the implementation of a navigation process to address the barriers to accessing mammography including using language and culturally-tailored messaging and media campaign. The “Study” stage is defined by tracking enrolled patients and evaluating program measures that include completion of screening and diagnostic services and, then, in the “Act” stage, optimizing the program to expand the reach of the program to serve greater proportion of un- and under-insured women.

### Development of a Breast Cancer Screening Navigation Program

In the “Plan” stage, the critical elements are: (1) securing the funding to establish a cost-free screening mammography program; (2) selection, training and defining the functions of the breast cancer screening navigator; and (3) establishment of a referral network of community partners that serve the health needs of un and under-insured women.

### Securing the Funding to Establish a Cost-Free Screening Mammography Program

The PBHI program costs include: (1) patient care costs for screening and diagnostic mammography, breast ultrasounds, office visits and biopsies which result from both professional and facility fees; (2) patient navigator salary, benefits and training costs; (3) program costs include routine administrative costs such telephone services, mail and postage, program promotional and educational materials, as well as the costs to overcome patient barriers including transportation and interpreters. A combination of contractual, grant and institutional funding covered the total costs of the program as described below.

In 2014, the ACC became a designated Pennsylvania Department of Health (DOH) Healthy Woman Program site. The Pennsylvania DOH Healthy Woman Program is funded by the CDC National Breast and Cervical Cancer Early Detection Program (NBCCEDP), a nationwide, comprehensive public health program with the mission of increasing access to breast and cervical cancer screening for women who are medically underserved. Established in 1990, one of the objectives of the program is to target the racial/ethnic disparities in screening, diagnosis, and treatment of breast and cervical cancers. The program provides funding in all 50 states, the District of Columbia, 6 US territories, and 13 American Indian/Alaska Native tribes or tribal organizations. Program sites are allocated slots based on the demonstrated need in their communities. Many large metropolitan cities have dozens of BCCEDPs to meet the needs of their large un- and underinsured populations. Of the 1,309,350 women undergoing breast screening or diagnostic services through NBCCEDP between July 2013 and 2018 across the U.S., almost 70% were from racial/ethnic minority groups (14). The NBCCEDP strategies to increase screening and breast cancer treatment among racial/ethnic minorities include reminders for patients, culturally-tailored programs that address specific beliefs or knowledge gaps, and programs addressing financial or logistical barriers to screening (15). An analysis of the NBCCEDP estimated that the number of life-years saved between 1991 and 2006 was 100,800 compared with no program and 369,000 life-years compared with no screening (16).

Through a combination of this contract and a portfolio of grants including from the Susan G. Komen Foundation and other foundations as well as institutional funding from the ACC, the program has been able to provide an increasing number of free breast screening services to eligible women. The ACC and the Rena Rowan Breast Center provided additional funding for personnel costs and Penn Medicine has provided support through office space and translation services.

By contract with the Pennsylvania DOH, Medicaid rates (and currently Medicare rates) were the maximum reimbursable rates. The PBHI established a billing contract with Penn Medicine that allowed the program to charge the Medicaid and Medicare-adjusted rates for all breast services provided by the PBHI (rather than commercial rates) which allowed the program an even greater capacity to support and serve the greatest number of women possible.

### Patient Navigator Selection, Training, and Functions

The PBHI navigator is a Masters of Public Health (MPH)-trained individual who acts as bridge between the community and the health care system. The PBHI navigator had to be proficient at creating community partnerships that permit the identification of un- and under-insured women in communities, lead and participate in community educational and outreach events and navigate patients through a complex health care system. The navigator received further training at the Harold P. Freeman Patient Navigation Institute in New York, New York, which focuses on teaching skills to maximize retention, diagnostic and treatment resolution rates, cancer navigation best practices and the conduct of navigation research (17).

The primary function of the navigator is to create a referral base from community partners and navigators and facilitate access to screening, diagnostic or treatment care by addressing language, literacy and cultural barriers, provide emotional support to patients to reduce fear and improve patient–provider communication. To overcome communication challenges posed by differences in language, culture and limited interactions with health care, communication via the referring community navigators and partners was determined to be most effective to establish contact with patients.

The navigator also coordinates the administration, financial and reporting responsibilities of the program. The navigator collaborates with radiology departments to implement the program's enrollment protocols into the registration and check-in process. In addition, the navigator also works with the financial department to create corporate guarantor accounts and prevent the direct billing of services rendered to patients. It is imperative to avoid directly billing patients which can create a record of debt for patients and even jeopardize their credit records. Finally, the navigator is responsible for maintaining detailed records to allow for call backs for screening or diagnostic testing when medically necessary, for administrative reporting to grant sponsors and for internal quality improvement and program evaluation purposes.

### Establishing a Referral Base From a Network of Community Partners That Serve the Health Needs of Un- and Under-Insured Women

Since un- and under-insured patients are unlikely to seek a screening mammography due to its prohibitive costs and lack of access, it was critical for the navigator to develop a robust network of community partners which serve the un- and underinsured communities in the region. Since a portion of the target population is already accessing primary care through Federally Qualified Health Centers (FQHCs), the PBHI partners with multiple primary care clinics at FQHCs and non-profit community organizations to identify and refer the target population for breast cancer screening services (Table 1). During the time this program was planned, over 300,000 patients made visits to Philadelphia area FQHCs and other community health centers with 97% of patients reporting incomes <200% of the Federal Poverty level (18). Subsequently, the program has established and maintained over a dozen partnerships with FQHCs in the Philadelphia area which have been critical in the development of the program's referral base and reaching the target population.

**Table 1:** Penn Medicine breast health initiative community partner network.

**Organization**	**Type of organization**	**Organization description**	**Location**	**Primary population served**
Health Promotion Council	Non-profit	An affiliate of Public Health Management Corporation (PHMC)	Center City West	Hispanic/Latinx
Southeast Asian Mutual Assistance Association Coalition (SEAMAAC)	Non-profit	One of the oldest and largest refugee founded agencies in the region	South Philadelphia	Asian
Congreso de Latinos Unidos	Non-profit, FQHC	FQHC in partnership with PHMC	Fairhill	Hispanic/Latinx
BEBASHI	Non-profit	Services for breast health, sexual health and hunger relief	Kensington	Black
Health Annex	FQHC	An affiliate of Family Practice & Counseling Network, a program for Resources of Human Development	Southwest Philadelphia	African, White, Black
National Black Leadership Initiative on Cancer (NBLIC)	Non-profit	Greater Philadelphia Chapter, launched as the first minority outreach project of the National Cancer Institute under the leadership of Louis W. Sullivan, M.D., Morehouse School of Medicine President Emeritus and Former Secretary of the US Dept of Health and Human Services	Poplar	Black
African Family Health Organization (AFAHO)	Non-profit community based organization	A non-profit organization that connects African and Caribbean immigrants and refugees to health care	Belmont	African and Caribbean immigrants and refugees
Puentes de Salud	Non-Profit	A non-profit focused on health, education and community building	Center City	Hispanic/Latinx
Greater Philadelphia Health Action (GPHA)	Non-profit	A non-profit to increase access to health care for un and underinsured families	12 locations throughout the Greater Philadelphia region	White, Black, Asian, Hispanic/Latinx
Maria de los Santos Health Center	Community Health Center	Part of Delaware Valley Community Health, Inc., the largest provider of primary health care services to Latinos in Philadelphia	Fairhill	Hispanic/Latinx
Norristown Regional Health Center	FQHC	Part of Delaware Valley Community Health, Inc., Montgomery County's first FQHC providing health, behavioral and dental services to communities	Norristown	Hispanic/Latinx, African American
Ludmir Center For Women's Health	Community Health Clinic	Based at Penn Medicine's Pennsylvania Hospital and a Healthywoman program provider for obstetrics and gynecology services	Washington Square/Center City East	Hispanic/Latinx
Rising Sun Health Center	Non-profit	Program of Philadelphia Health Management Corporation	Olney	Hispanic/Latinx
Cambodian Association of Greater Philadelphia	Non-profit	Philadelphia's foundation of social, health and education programs	Olney	Cambodian
Horizon House Health Center	Primary Care Center	First behavioral health care center in Philadelphia to integrate medical care and behavioral health	Fairmount	White, Black, Asian, Hispanic/Latinx
Philadelphia Corporation for Aging (PCA)	Private, non-profit	One of the largest non-profit organizations in the region	Fairmount	Seniors, people with disabilities

On the other hand, many un-and underinsured women are not accessing care at all. To reach this portion of our target population, the PBHI partners with community-based, non-profit organizations that employ community lay navigators that can identify and refer patients to the program. One example of such a partner organization is the Health Promotion Council of Southeastern Pennsylvania (HPC), an affiliate of the Philadelphia Health Management Corporation, a non-profit corporation that conducts community-based outreach, education and advocacy for vulnerable populations. Through a subcontract funded by the Susan G. Komen National Foundation, HPC employed two community navigators that helped to identify women in need of breast cancer screening in the community and linked these women with the PBHI navigator. These agencies organize many different free community events which helps the HPC community navigators identify women overdue for breast cancer screening and connect them with the PBHI navigator. The navigator also attends and leads outreach and educational events, health fairs and breast symposiums regularly to engage women in need of breast health services. The navigator follows up with these women by phone to enroll patients in the program and schedule screenings.

The navigator also receives referrals from word of mouth, as a result of marketing and advertising efforts and through internal daily requests via other Penn Medicine health care providers and employees such as patient service associates, radiology managers, when patients are deemed un- and underinsured. Referrals are made through a HIPAA-compliant form containing patient's contact information that is securely faxed to the navigator.

### Establishing a Navigation Process to Address the Barriers to Accessing Mammography

We developed a step-by step process for breast cancer screening navigation (Appendix 1) to outline the “Do” component of the PDSA cycle. The navigator must first confirm patient eligibility for enrollment in the PBHI. Patients must be of the guideline-recommended screening age and due for a screening mammogram or be experiencing a new breast problem, such as a breast lump, nipple discharge, breast mass or breast pain. To be enrolled in the Healthy Woman Program, patients must be a Pennsylvania resident, un-insured or under-insured (i.e., have a high co-pay or deductible they cannot afford) and meet household income guidelines (at or below 250% of the Federal Poverty Income level).

Un- and underinsured patients who are ineligible for the Healthy Woman Program and in need of breast services are enrolled in the PBHI and their services are covered using alternative grant or philanthropic funding. The additional funding also allows the program to serve New Jersey residents who are within the ACC's catchment area, but do not qualify for the Pennsylvania-funded program and to males experiencing a breast problem.

Over half (58%) of the PBHI's patient population speaks a language other than English (Table 2). To bridge language and communication barriers, the navigator has a dedicated phone line that patients can contact directly and uses a hospital-provided translational service when communicating with non-English speaking patients over the phone. In-person language interpreters and on-demand video interpretation via Martii devices are available to non-English speaking patients at their appointments. To further address language barriers, program promotional materials and breast health educational materials are available in a variety of languages including Spanish, Mandarin, and Vietnamese. Result and reminder letters are also translated for non-English speaking patients.

**Table 2:** Demographics (*n* = 1,974).

**Age, mean (s.d.)**	**48 (9.7)**
**Age distribution**	
<40	15%
40–49	45%
50–64	37%
65+	3%
**Race**	
White	44%
Black	27%
Other	17%
Unknown	12%
**Ethnicity**	
Hispanic/Latino	53%
Non-Hispanic/Latino	36%
Unknown	11%
**Language**	
English	42%
Non-English	58%
Spanish	78%
Mandarin	11%
Other	11%

Likewise, cultural barriers are prominent among the PBHI population and important to address. Differences in cultural beliefs between the provider and the patient affect how patients perceive medical information and the concept of health and illness in general. The PBHI navigator works closely with our diverse group of partner organizations to provide culturally-tailored messages and appropriate care. To improve cross–cultural communication, the navigator expresses any patient concerns to the point of contact from the patient's referring organization and seeks guidance about how to more effectively communicate with patients in light of differences in cultural beliefs.

Language and cultural barriers are linked with low health literacy (19). In addition to having program and educational materials in various languages, the PBHI ensures that all information is written for individuals with lowest literacy levels (6–8th grade) in order to effectively reach our target population. The navigator also offers patients help with completion of any forms needed to access care.

Transportation was also reported as a barrier by PBHI patients. To address transportation barriers, the PBHI offers public transportation vouchers to help patients and their caregiver travel to and from appointments.

Patients frequently report fear of a cancer diagnosis as a barrier in accessing care, especially after receiving an abnormal mammogram result. The PBHI navigator responds to statements about fear with information, emotional support and encouragement to patients. The navigator also provides patients with her direct land and cell phone numbers which allows patients to reach the navigator at their convenience if they had any questions or concerns.

In an effort to best accommodate patients, minimize travel and maximizing convenience, the program's services are available to patients at a variety of Penn Medicine's community radiology locations in the Greater Philadelphia region including Pennsylvania Hospital (Center City), The Perelman Center (West Philly), Radnor Hospital, Valley Forge, Bucks County, Woodbury Heights, NJ and Cherry Hill, NJ.

Furthermore, in addition to offering cost-free breast cancer screening services, the navigator offers referrals for free cervical cancer screenings at the Ludmir Center for Women's Health (LCWH), a Penn Medicine, Pennsylvania's DOH Breast and Cervical Cancer Early Detection Program/HealthyWoman program provider located at Pennsylvania Hospital, that offers gynecology and obstetrics services. This partnership involves close communication between the PBHI navigator and LCWH staff to ensure that new and existing un- and underinsured patients are receiving comprehensive women's health services.

One of the most important functions of the navigator and a measure of success of a breast cancer screening navigation program is follow up of patients with abnormal results to assure that they receive the necessary follow up diagnostic testing. The navigator has a detailed protocol for following up patients with abnormal results, that begins by contacting the program co-director via the electronic record (EPIC) with the imaging result and schedules further radiologic workup or an office visit for each patient. The navigator also generates daily work queues from EPIC to identify patients enrolled in the program who have abnormal screening results. The radiology departments also have a dual process for following up abnormal findings that also applies to the patients of our program. This includes an attempt to call the patient, a mailed letter notifying the patient to return for additional imaging with a telephone number to schedule the appointment, a reminder letter after 15 days as well as a second reminder letter and a letter to the referring physician after 30 days. If still unsuccessful in reaching the patient after six attempts, the navigator reaches out to the referring clinic, provider or community partner to ensure that the patient is not lost to follow up. For example, the PBHI partners with community navigators at the HPC who are available to visit patients' homes, if needed, to provide further education and explain the importance of follow-up studies to patients.

Patients who complete a diagnostic mammogram receive the results immediately after their test directly from the radiologist reading the studies, using translation services if necessary. Patients that require a biopsy are scheduled for appropriate follow-up studies immediately after receiving their results, and, if needed, arrangements are made so that they are accompanied by our partners' community navigators. This removes any uncertainty regarding appropriate follow-up and contact.

If a woman is diagnosed with breast cancer, the PBHI also offers assistance with enrolling women in the state health insurance plan. Immediately preceding a patient's office visit with a new diagnosis of breast cancer, the navigator initiates the first steps in obtaining coverage to minimize delays in receiving treatment. The navigator works with the Pennsylvania County Assistance Offices, chooses the office that is in closest proximity to the patient's home and gathers all the necessary documents that are needed to produce an effective application for obtaining coverage. Upon diagnosis, women are enrolled in the State Breast and Cervical Cancer Treatment Program and, if ineligible, for example if the woman is undocumented, then she is enrolled in Emergency Medical Assistance. The navigator also makes additional referrals to the ACC's Financial Advocacy team that determines if the patient may be eligible to enroll in a health insurance plan through the marketplace. After securing health coverage, the navigator transitions the patient into treatment at the ACC where the Initiative's co-director, and the ACC's team of cancer specialists and nurse navigators continue to support the patient throughout their treatment.

### Developing Culturally-Tailored Messaging and a Media Campaign

Another important component of the “Do” stage in developing this program was the development of a multilingual messaging and media campaign tailored to the needs of the diverse patients served by the program. According to the Pew Charitable Trusts' Philadelphia Research Initiative, in the last 10 years there has been significant growth in Hispanic communities in Philadelphia (20). Moreover, 319,310 of Philadelphia residents speak a language other than English with the most common foreign language being Spanish (20). Limited English proficiency is a major barrier in the delivery of medical care among the uninsured: women who speak Spanish are less likely to be screened for breast cancer (21). In an effort to develop culturally-tailored messaging to decrease breast screening disparities among the Latina population in our community, the PBHI partners with the American Cancer Society and Univision 65 to hold an annual, “Amate a ti Misma” or a “Love Yourself,” campaign to encourage women to undergo an annual screening mammography. The campaign messages, delivered in Spanish by Univision newscasters, focuses on an all-day screening event located at the Pennsylvania Hospital. The message addresses the prevalent cultural beliefs in Latin cultures rooted in Marianismo, specifically Familismo, that encourage women to care for other members of her family and dissuade Latinas from putting their needs first. The message asks Latinas to love themselves by undergoing a mammogram. The month leading up to the event, Univision airs public service announcements which include the PBHI navigator's phone number. Patients interested in attending call the navigator to schedule an appointment. If women are unable to attend the event, they are scheduled for another more convenient day and time.

### Program Evaluation and Optimization

Patient navigation process measures are carefully captured by the patient navigator to allow us to “Study” the program and then “Act” to more effectively and efficiently accomplish the goals of the program. The program tracks the number of patients referred for services, appointments made and kept, type of service(s) rendered and their date, screening results, follow up recommendations, demographic information (race, ethnicity, age, zip code, city and state, insurance status), referral information (for breast cancer screening and treatment services and for cervical cancer screening), navigation services received, and barriers addressed (including transportation and language support), among others (this data not shown). The Initiative also tracks patient outcomes including mammogram and biopsy results, time to diagnostic resolution, cancer diagnoses, stage of cancer at diagnosis and cancer treatment (some of this data is discussed below and shown in Tables 3, 4).

**Table 3:** Mammography results by breast imaging reporting and data system (BI-RADS) category (*n*-1,974 unique patients).

**BI-RADS**	**Screening mammogram (*n* = 1,761)**	**Screening mammograms with incomplete follow-up (*n* = 11)**	**Diagnostic mammogram (*n* = 654)**	**Diagnostic mammograms with incomplete follow-up (*n* = 31)**
**Cat 0** – Incomplete Exam	167	7	16	5
**Cat 1** - Negative	1,194	–	123	–
**Cat 2** – Benign	363	–	287	–
**Cat 3** – Probably Benign	15	4	118	22
**Cat 4** – Suspicious	19	–	95	4
**Cat 5** – Highly Suspicious	3	–	15	0

**Table 4:** Stages of breast cancer diagnosed (*n* = 25).

**Stage**	***n***
0	3
I	6
II	7
III	6
IV	3

Finally, patients enrolled in the HealthyWoman program are required to complete the Pennsylvania DOH HealthyWoman Program enrollment forms before appointments. Patients complete the forms at check-in and the radiology check-in staff is required to fax the forms to the navigator and scan the form in the patient's electronic medical record. The navigator later enters all forms into the DOH's data management system, Med-IT, a requirement for reimbursement of services rendered. Med-It is a web-based health screening information database system that includes demographic information, automatic eligibility computation, billing and much more. The navigator is able to query the Med-IT database for clients by provider reports, billing reports and demographic reports, which allows the evaluation of the HealthyWoman program's data separately.

The Initiative measured patient satisfaction in an initial subset of 90 patients that enrolled in the program using an adapted, previously published instrument (22). The adapted instrument which was made available via a translator in the patient's native language, consists of 9 items with a 5-point Likert response scale with anchors very satisfied to very dissatisfied. The mean scores for each of the 9 items ranged from 4.68 to 4.95 out of a maximum of 5.00 indicating that patients were generally very satisfied with the services offered by the program (Table 5).

**Table 5:** Patient satisfaction with patient navigation services (*n* = 90)^*^.

**For each problem, indicate whether you were very satisfied (very happy), a little satisfied (happy for the most part), or not satisfied (not happy) with the help you received from your navigator(s)**.	**Score**
1. Making medical appointments	4.95
2. Getting results of tests you had	4.80
3. Dealing with financial concerns related to getting the care you need	4.84
4. Getting transportation to the doctor's office	4.72
5. Giving you emotional support	4.86
6. Dealing with fears related to your health issues	4.91
7. Getting the health information you need	4.88
8. Understanding the medical tests you got	4.68
9. Dealing with doctors, nurses, and other health care workers who do not speak your language	4.92

* *Adapted from Patient satisfaction with logistical aspects of navigation (PSN-L) scale (20)*.

The ability to capture and analyze all of these measures allowed us to reflect and innovate (“Act”) to optimize the program and more effectively and efficiently reach its goals of increasing access to screening and diagnostic mammography and ultimately reduce the burden of breast cancer among un- and under-insured women. Our multidisciplinary team met regularly to map and measure the program's processes, identify barriers to accessing the program and redefine our work. The PDSA model allowed us to strengthen the program over time by allowing us the ability to reflect on populations that we were not able to initially reach and then adjust the program to better serve those populations. For example, initially, there were women who needed screening or diagnostic mammography, but who did not meet the eligibility for the Pennsylvania DOH Healthy Woman Program because they fell out of the eligible age range and resided in an adjacent state. This led our team to apply for a Susan G. Komen grant that allowed us to serve these women. In another example, the PDSA model allowed us to recognize that the program was not fully reaching Asian women. Consequently, we sought to develop additional partnerships with community organizations that serve the Asian community and grant support to expand our outreach and engagement with this community. Below we describe some of the program's results and key successes that resulted from the iterative application of the PDSA cycle to the PBHI as well as program challenges.

## Results

A key success is that over the past 5 years since the program's inception (June 2014–June 2019), the Initiative has navigated 1,974 racially and ethnically diverse women providing over 2,000 high quality breast services to women who were previously unable to access mammography (Table 2). Table 2 also shows the diversity of the race and ethnicity of the women enrolled in the program: 10% Asian, 27% Black, 7% other, 12% Unknown, 44% White; 53% Hispanic. Over half (58%) of women who participate in the program speak a language other than English, with 78% Spanish as the most common, 11% speak Mandarin; 11% speak other languages. Similar to prior research, this work shows that breast cancer screening navigation programs for uninsured women are effective and lead to increased screening rates and the detection of previously undetected breast cancers (23).

Table 3 demonstrates that among 1,974 unique patients, the program delivered 1,761 screening and 654 diagnostic mammograms. Among these 11 patients who completed screening mammograms and 31 patients who completed diagnostic mammograms did not complete follow-up diagnostic images. Despite multiple attempts by our navigator by telephone and mail, via the Health Promotion Council Navigators and the radiology providers and/or staff, these women were lost to follow up. In a few of these cases, family member or community partners or navigators confirmed that the woman refused additional images, sought care elsewhere or returned to her native country, but the reason for incomplete studies was unknown for the majority of patients and they were classified as lost to follow up.

Another important success of the program is the diagnosis of breast cancer in 25 patients - all of whom were enrolled in health insurance and treated at the ACC (Table 4). The majority of these were diagnosed at a localized stage and that all patients have received high quality treatment at and support from an NCI-Comprehensive Cancer Center.

As shown in Table 5, yet another success is the high patient satisfaction reported among an initial subset of women enrolled in the program which was measured using the Patient Satisfaction with Logistical Aspects of Navigation Scale (20). However, this subset represents a group that was slightly older and had half the proportion of Hispanic patients than the larger cohort we report here (data not shown). Therefore, the satisfaction results may not be generalizable to the results of the cohort reported here.

## Discussion

For the over 28 million Americans without health insurance (8), hospitals often represent their only source of care. Consequently, there is a great need to develop programs that help meet the health needs of the uninsured populations and deliver high quality care. We describe the development of a program that could be replicated to meet the breast cancer screening needs of un- and under-insured women. The application of the Plan-Do-Study-Act quality improvement framework to the development, implementation, and optimization of a breast cancer screening navigation program for racially and ethnically diverse un- and under-insured women and outline the critical elements to guide this work. Among the critical steps ascertained through this process are: procurement of program funding from the DOH, grants and philanthropic sources; training of a navigator in the establishment of a referral network of community partners that serve the un and under-insured; establishment of a navigation process that can overcome barriers to screening and care; design a language and culturally-tailored messaging and media campaign; and establishment of clear process measures and evaluation that can further inform the optimization of the program.

The Penn Medicine Breast Health Initiative was created using this framework and this analysis summarizes the program's reach and impact. In the first 5 years of this program, it reached almost 2,000 women and identified 25 cases of breast cancer. Among the 25 women, only three were identified at stage 4. All 25 women were enrolled in health insurance and provided with high quality care at an NCI comprehensive cancer center. Patient satisfaction with the program, measured in a small subset of 5% of patients, was high.

The major challenges to establishing a breast cancer screening navigation program for un- and under-insured diverse patients include securing the funding for the program's operating support which includes the navigator salary, benefits and training which can be secured through grant, philanthropic and/or institutional funding. There are also logistical challenges to reaching this difficult-to-reach population both before engaging in the program and after for follow up care. This population generally has transient phone and sometimes housing access. Traditional methods of communication by telephone and mail fail these individuals. During situations where patients have an abnormal finding and require additional follow up, the navigator works with the referring center that women trust and, at times, community navigators, to locate the patient. Potential future strategies for reaching women might include contacting them at their place of employment or other faith-based and non-faith based community organizations. There is also the challenge of securing insurance for patients in a timely manner once they are diagnosed with cancer. This challenge can be overcome assigning a case worker or financial advocate to the patient and having this individual work closely with the navigator who already has an established relationship and trust with the patient. Furthermore, under the Protecting Access to Medicare Act of 2014, in order to expedite insurance coverage and obtain access card information for any patients enrolled in emergency medical assistance, the navigator must also complete a waiver to allow the navigator to retrieve the patient's coverage information on the patient's behalf.

Despite these challenges, the BCCEDP program provides funding to meet the breast cancer screening needs of un- and under-insured women. We have demonstrated that the Plan-Do-Study-Act quality improvement framework can be used to develop, implement, and optimize a breast cancer screening navigation program that can identify and reduce the burden of breast cancer in women at risk.

## Data Availability Statement

The raw data supporting the conclusions of this article will be made available by the authors, without undue reservation.

## Ethics Statement

The studies involving human participants were reviewed and approved by University of Pennsylvania Institutional Review Board. Written informed consent for participation was not required for this study in accordance with the national legislation and the institutional requirements.

## Author Contributions

All authors take responsibility for the manuscript, have directly participated in the planning, development, writing of the article, read and approved the final version, and consent to our names on the manuscript. CG have full access to all aspects of the review and writing process and take final responsibility for the paper.

## Conflict of Interest

The authors declare that the research was conducted in the absence of any commercial or financial relationships that could be construed as a potential conflict of interest.

## Abbreviations

ACC: Abramson Cancer Center
FQHC: Federally Qualified Health Center
HPC: Health Promotion Council
DOH: Department of Health
LCWH: Ludmir Center for Women's Health
MPH: Master of Public Health
NBCCEDP: National Breast and Cervical Cancer Early Detection Program
PBHI: Penn Breast Health Initiative
PDSA: Plan, Do, Study, Act.
