# Supplementary material for: A Plan-Do-Study-Act Approach to the Development, Implementation and Evaluation of a Patient Navigation Program to Reduce Breast Cancer Screening Disparities in Un- and Under-Insured, Racially and Ethnically Diverse Urban Women
Source: Front Public Health. 2021 Feb 19;9:595786. doi: 10.3389/fpubh.2021.595786 (PMC7933216; doi:10.3389/fpubh.2021.595786)
Supplement: Supplementary file 1 [file Data_Sheet_1.pdf]

Appendix: Step by step process for breast cancer screening navigation

1.) **Patient Referrals:**

- The navigator receives patient referrals from…
  - the PBHI's community partners (fill out our referral form and fax to navigator)
    - The navigator will review the referral form and determine what service is best needed for patient, to gain an idea of what is needed prior to speaking with the patient and to decide if an order is needed.
  - community outreach and education events (fill out referral from & contacts when back on site)
  - marketing and advertising (small and mass media campaigns)
  - word of mouth
  - internal referrals (via UPHS employees when patients are deemed uninsured and underinsured)
- The navigator then confirms the patient's language. If the patient speaks a language other than English, the navigator uses the Quantum language interpretation line to communicate with the patient.
- The navigator then attempts to contact the patient and if the navigator is unable to reach the patient, the navigator will leave a voicemail with the call back phone number where she can be reached. The navigator will make 4 – 6 attempts to contact the patient.
- If the navigator is having a difficult time reaching the patient, she reaches out to the partner organization from where the partner was referred from and they work together to reach the patient.

1a. Eligibility:

- The navigator confirms patient eligibility requirements:
  - ✓ Insurance status: no health insurance or under-insured (high co-pay or deductible they cannot afford)
  - ✓ Meets recommended screening age guidelines: (40 – 64 years of age) & is due for a mammogram

    OR
  - ✓ Patient is experiencing new breast problem: Regardless of age (new breast lump, mass, nipple discharge, and/or localized pain)
  - ✓ Residency:
    - If the patient lives in Pennsylvania → enrolled in PA DOH's HealthyWoman Program
    - If the patient lives in New Jersey → supplementary grant funding is used to cover the patient's service
  - ✓ Income: Women whose household income is at or below 250% of the Federal Poverty Income Guidelines are eligible.
- If the patient does not fit the eligibility requirements described above, the navigator uses supplementary grant funding to cover the patients' services.

2.) **Education & setting up appointments:**

- Once eligibility is confirmed, the navigator begins the scheduling process with the patient.
- The navigator will schedule any of the following as determined by factors such as symptoms and age:
    1.) Screening mammogram
    2.) Diagnostic Mammogram
    3.) Ultrasound
    4.) Biopsy
    5.) Office Visit
- In addition to facilitating access to care, the navigator ensures it is patient centered, meaning unique to each individual patient by helping address any barriers that may exist.
- The navigator educates patients about breast cancer screening and assists each patient with scheduling an appointment at one of seven of Penn Medicine's community radiology locations or for an office visit, if needed.

3.) Addressing patient barriers: The navigator identifies and addresses each patient's individual set of barriers.

- Health insurance coverage/cost – (Not having health insurance or having a health insurance plan with a high co-pay or deductible they cannot afford) The PBHI eliminates cost as a barrier by providing free mammograms and other breast diagnostic services to women who are un- and underinsured.
- Language - If the patient speaks a language other than English, the navigator uses the Quantum Inc. interpreter language line to speak with the patient. In person language interpreters and MARTI video interpreters are available at appointments if needed.
- Cultural – The PBHI partners with organizations and clinics that serve diverse women in medically underserved areas. The navigator works closely with each partner's point of contact such as their outreach worker, community navigator, case manager, etc. to ensure that care is culturally competent. The PBHI partners with Spanish media television to create culturally-tailored messages that address prevalent cultural beliefs in Latin cultures rooted in Marianismo, specifically Familismo, that encourage women to care for other members of her family and dissuade Latinas from putting their needs first. The message asks Latinas to love themselves by undergoing a mammogram.
- Education and Health Literacy - The program's promotional materials and educational materials are available in 6 languages. The navigator addresses patient questions and concerns. The PBHI offers one on one breast education sessions, group breast symposiums, and breast health presentations.
- Difficulty reaching the patient – Collaboration with community partners and their clinical staff to reach the patient. The navigator has a dedicated phone during normal

work hours and a work cell phone for patients to contact after business hours during the week or on weekends.

- Transportation – offers bus/train tokens to help patients get to and from appointments
- Fear & Distrust – provide emotional support and meet all the patient's needs

4) Addressing clinical results

- If the mammogram is normal, then the navigator sends a reminder to the patient to return in 1 year
- If the mammogram is abnormal, then the navigator will schedule biopsy or office visit with breast surgeon based on what is needed.
- If the biopsy is normal, then the navigator sends a reminder to return as recommended by the clinician.
- If the biopsy reveals breast cancer, then the navigator enrolls patient in health coverage (BCCPT or EMA) and refers to the cancer treatment navigator
